# Parents' Experiences of Psychosocial Support in the Neonatal Intensive Care Unit: A Qualitative Study

**DOI:** 10.1111/jocn.70417

**Published:** 2026-06-25

**Authors:** Hanna Seppänen, Eeva Talus, Ulla Sankilampi, Krista Koivula, Ilona Luoma, Tarja Pölkki

**Affiliations:** ^1^ Research Unit of Health Sciences and Technology University of Oulu Oulu Finland; ^2^ Finland Medical Research Center Oulu Oulu University Hospital and University of Oulu Oulu Finland; ^3^ The Finnish Centre for Evidence‐Based Health Care: A Joanna Briggs Institute, The Finnish Centre of Excellence Helsinki Finland; ^4^ Institute of Clinical Medicine University of Eastern Finland Kuopio Finland; ^5^ Department of Paediatrics, Child and Adolescent Center of Excellence Kuopio University Hospital Kuopio Finland; ^6^ Department of Child Psychiatry, Child and Adolescent Center of Excellence Kuopio University Hospital Kuopio Finland

## Abstract

**Aim:**

To explore parents' experiences of psychosocial support in the neonatal intensive care unit (NICU).

**Design:**

A qualitative descriptive study.

**Methods:**

The study involved nine individual and three pair interviews; in total, 15 parents described their experiences of psychosocial support in the neonatal intensive care units. The data were analysed using inductive content analysis.

**Results:**

Parents' experiences of psychosocial support consisted of four main categories: (1) supporting parenting; (2) strengthening the sense of security; (3) social support; and (4) mental wellbeing support. Parents described how important it was to receive support in caring for an infant, to be able to participate in decision‐making or to feel safe despite a difficult situation.

**Conclusion:**

Even though parents described peer support and trauma‐informed care as important forms of support, many NICUs lacked these. The results provide essential information to healthcare professionals, enabling them to understand and support parents. This can increase parental satisfaction and improve the wellbeing of families, as well as support parents during neonatal intensive care and after discharge.

**Implications for the Profession and Patient Care:**

This study provides tailored information on parents' experiences of psychosocial support in neonatal intensive care units. The results can be used in the education of healthcare professionals and to assist them in integrating psychosocial support methods into their practice, such as consistent emotional support.

**Impact:**

The findings emphasise the importance of systematically asking parents about their experiences in order to identify their needs and the various forms of psychosocial support they require. These insights can guide healthcare professionals in providing personalised psychosocial support to parents in NICUs.

**Reporting Method:**

The reporting was guided by the Consolidated Criteria for Reporting Qualitative Research (COREQ) checklist.

**Patient or Public Contribution:**

Parents contributed to the study through data collected from the national peer support association (Kevyt) in Finland.

## Key Points

- What does this paper contribute to the wider global clinical community?
  ○ The importance of asking parents about their experiences in order to identify their needs and the different forms of psychosocial support they require.
  ○ Understanding how tailored psychosocial support can strengthen parents' sense of security.
  ○ Knowledge to guide healthcare professionals in providing personalised psychosocial support in NICUs.

## 1 Introduction

Globally, almost 30 million families have a sick or premature infant needing treatment in the neonatal intensive care unit (NICU) annually (World Health Organization 2018). The NICU environment is psychologically stressful for parents, and as much as 40%–50% of parents experience symptoms of traumatic stress, anxiety and depression (Lakatos et al. 2019; Segre et al. 2014). Parental stress in the NICU is a worldwide issue (Caporali et al. 2020), and it can disrupt parenting, early parent‐infant interaction, and it can have negative impacts on participation in the care of the infant and other children in the family (Yaman and Altay 2015). Parents require immediate and sufficient support to mitigate adverse effects and enhance their wellbeing in the NICU (Caporali et al. 2020). Therefore, there is an urgent need to identify parents' experiences of psychosocial support to avoid these adverse effects for parents of infants requiring intensive care.

## 2 Background

Psychosocial support includes all activities that are organised to reduce and alleviate the effects of psychological stress caused by trauma (National Institute for Health and Care Excellence 2018). Psychosocial support encompasses support from healthcare professionals, as well as support from family and the individual's own resources (WHO 2020; NICE 2018). Psychosocial support also includes peer‐to‐peer support, which offers shared experience and can be delivered through hospital‐ or peer‐support association‐based programs, including person or telephone matches and support groups that meet in person or via the Internet (Hall et al. 2015). WHO (2022) states that psychosocial support benefits all mothers in postnatal care, but especially mothers with mental symptoms or risks.

The need for an infant's care in the NICU causes psychological distress for parents (van Wyk et al. 2024), and symptoms can be long‐lasting. As many as one‐third of parents have posttraumatic stress disorder symptoms 1 year after discharge (McKeown et al. 2023). The NICU environment itself is stressful for many parents (Adama et al. 2022) and the severity of the infant's illness, birth weight and prematurity can also affect the burden experienced by the parents (van Wyk et al. 2024). Parents who experienced more uncertainty were more likely to be diagnosed with perinatal post‐traumatic stress disorder after the infant's discharge from the NICU (Malin et al. 2022).

Some factors that may increase parents' psychological burden have been identified. The experiences of mothers and fathers are partly similar, but there are also differences that should be considered. Mothers report psychological distress more than fathers. Mothers' stress symptoms are primarily related to their relationship with the infant, such as parental role or separation from the infant, and the inability to take care of the infant or to prevent the infant's pain (van Wyk et al. 2024). Infants requiring intensive care increase the mothers' risk of developing posttraumatic stress disorder (Sharp et al. 2021) or postpartum depression (Shovers et al. 2021), which can have adverse effects on mothers' mental health and interactions with the infant, spouse and other close family and friends (Slomian et al. 2019). The infant's need for intensive care also causes psychological distress to the fathers, but there is less research on this topic. Paternal stress symptoms are primarily related to the relationship with the infant, such as the inability to participate in infant care, not being involved in decision making, and lack of bonding or visiting (van Wyk et al. 2024).

Some previous studies have described parents' experiences of being in the NICU by interviewing (Loewenstein et al. 2019) and the support interventions for parents may reduce parental anxiety, depression, stress and posttraumatic stress syndrome in the NICU (van Wyk et al. 2024). There are still identified gaps in the current understanding of parental support and models of NICU care, which require cooperation with the parents. Many studies focused only on preterm and low birthweight infants and their families (Franck and O'Brien 2019). Psychosocial support, such as maternal acupuncture, relaxation or music interventions for parents, may reduce negative effects (Lakatos et al. 2019), but more research is needed because the optimal combination of psychosocial support is unknown (van Wyk et al. 2024). Significant gaps have been identified in parent support services, despite the recognition of parental distress (Ginsberg et al. 2023).

## 3 The Study

### 3.1 Aim and Research Question

This study aimed to explore parents' experiences of psychosocial support in the neonatal intensive care unit. The research question was as follows: What types of psychosocial support did parents experience during their infant's intensive care?

## 4 Methods

### 4.1 Design

This was a qualitative descriptive study design (Kyngäs et al. 2019), as the study aimed to explore the experiences of the interviewees and to present the experiences accurately. The reporting was guided by the Consolidated criteria for reporting qualitative research (COREQ) checklist (Tong et al. 2007).

### 4.2 Study Setting and Recruitment

The study invitation was published on the social media pages of the national peer support association (Kevyt) in Finland. The study used purposive sampling. The inclusion criteria for the study were being a parent of an infant who required neonatal intensive care less than 2 years ago, the infant's discharge from the NICU and the parent's ability to speak Finnish. The exclusion criteria were ongoing neonatal intensive care and the parents' age under 18 years. The study invitation included a link for parents to express their willingness to participate in the study. After that, the researcher contacted the parents to arrange an interview time. Two of those who had expressed their desire to participate in the study were not reached. The researcher verified the parents' suitability for the study when arranging the interview time and one parent was excluded because it had been over 2 years since the infant was discharged from the NICU. If both of the infant's parents agreed to participate in the study, they could choose an individual or a pair interview.

The parents who participated in the study received care for their infants at either a university hospital or a central hospital in Finland. University hospitals typically treat infants born significantly prematurely and those who are more seriously ill, whereas central hospitals generally care for infants in better condition. There is some variability in psychosocial support across NICUs in Finland, and the methods currently vary between hospitals. At the time of the study, the single‐family room model had been implemented in all but one hospital. Several hospitals had a multidisciplinary early interaction team (Vavu) which aimed to strengthen parent‐infant interaction. Some hospitals also had a social worker and mental health services, while a few had a crisis worker.

The first author (H.S.) was aware that her background as a paediatric nurse could potentially influence the interviews and the analysis of the results. However, this also helped the researcher to understand the phenomenon and to sensitively approach the parents.

### 4.3 Data Collection

Parents were interviewed using individual (*N* = 9) or paired (*N* = 3) unstructured interviews to accurately describe their experiences (Kyngäs et al. 2019). The researcher conducted online interviews with parents (*N* = 15). One open‐ended question was used: What kind of psychosocial support did you receive during the infant's intensive care? In addition, the interviewer used *follow‐up questions*, such as: What kind of professional groups visited you, and what kind of support did you receive from them, how your family was supported during the infant's intensive care, and what kind of information/guidance made you feel supported.

Before the interviews, the researcher conducted one interview to test the equipment and the interview questions. This interview was excluded from the data because more than 2 years had passed since the child was discharged from the NICU. No changes were needed to the interview questions or equipment based on the pilot interview. The interviews were collected over a 3‐month period (December 2022–February 2023). The interviews were recorded with the parents' permission, and parents were allowed to choose whether to keep the camera on or not. The average duration of the individual interviews was 67 min (range 38–115 min) and in pair interviews 81 min (range 66–98 min). The interviews were transcribed verbatim (216 pages, Times New Roman, font size 12, line spacing 1), and their accuracy was checked by comparing the transcript with the recording.

### 4.4 Data Analysis

The data were analysed using inductive content analysis due to the fragmentation of previous research data and to highlight the individual experiences of parents (Elo and Kyngäs 2008). In the preparation phase, the analysis of the data began with reading the data several times (H.S.), which enhanced credibility. A meaningful unit was chosen as the unit of analysis and during the content analysis, original expressions and sentences that were related to the study objective were identified. In the organisation phase, the data were used to find original expressions that correspond to the research question. The original statements (*N* = 357) were reduced (H.S.) by condensing them into shorter formulations while preserving their essential meaning and removing content irrelevant to the research question. The success of the reductions was assessed by two researchers (H.S., E.T.). Those reductions were then classified into subcategories (*N* = 39), and the subcategories were combined into categories (*N* = 10) and finally main categories (*N* = 4) (authors H.S., E.T. and T.P.) (Kyngäs et al. 2019) by following a systematic approach, and dependability was ensured. In cases of discrepancy, the researchers discussed their views until a consensus was reached. An example of the progress of the analysis of the data from original expressions to subcategories is described in Table 1. In the reporting phase, the results were presented in categories, illustrated with original expressions that support the study's confirmability; these categories are described in Table 2. The analysis was conducted manually without the use of qualitative data analysis software.

**TABLE 1:** An example of the progress of the analysis of data from original expressions to subcategories.

Original expressions	Reduced meaning unit	Subcategory
We had a good situation because (child's name) is our first child. We got the dog to be taken care of by the grandparents, and we had nothing but time. When there are such families that there are older siblings and we live 200 km away. When there is quite a lot more to do in that everyday life than the little premature baby that we were able to fully focus on. 6F	The grandparents helped by taking care of the family dog while the parents were with the infant.	Support in daily routines by family and friends
Of course, my loved ones helped, because my parents took good care of our older ones (children) so of course it helped me cope in that regard. 11M	Grandparents took care of the other children in the family	
I got so much help from my mother that somehow, she was there to support me. 7M	My own mother's help	
My friends bought us meal tickets and coffee tickets to the hospital. And then they brought us a surprise bag for the baby there. They were the best, because when I'm so careful about money, I had to go and use them because I was terribly stressed if I didn't use them during this hospital time. So, they were like the best, because they made us leave that room and eat well. 2M	Meal tickets bought by friends made her take care of her own meals	
	Meal tickets bought by friends made her leave the room for a while	
And so do my loved ones. We got food deliveries to our homes and goods deliveries to our homes, so we didn't have to go to any shops and stuff like that. 6M	Family and friends brought food for parents	
	Family and friends bought necessary stores for parents	
Then I had my friends and family again, who kind of said that now I should go for a walk myself. 7M	Family and friends said that it would be worth going for a walk	

**TABLE 2:** Categories based on inductive content analysis of the parents' experiences of psychosocial support in the Neonatal Intensive Care Unit.

Subcategories	Categories	Main categories
Preparing parents in advance for admission to the NICU	Support from healthcare professionals at different stages of care	Supporting parenting
Healthcare professionals support during an infant's care		
Preparing parents for infant discharge		
The calming space for parents in the NICU	Environmental support for parental presence	
The parents' possibility of privacy		
Implementing a single‐family room model		
An environment that considers the needs of parents		
Guiding parents about the NICU environment		
Parental participatory practices and working methods	Increasing parental participation in the care	
Parents' opportunity to participate in care planning and decision making		
Considering parents' wishes regarding care		
Compassionate behaviour of nurses and doctors	Emotional support	Strengthening the sense of security
Nurses' interaction skills		
Parents` feeling that the family is well taken care of		
The same nurses are taking care of the infant	Safety‐enhancing practices	
Possibility to get additional support for infant care if needed		
Clear division of responsibilities between healthcare professionals and parents		
Healthcare professionals had time for parents		
Competent healthcare professionals		
Sharing an experience with peers	Peer support	Social support
Participation in peer‐support groups		
Peer support on the internet		
Getting contact information for a peer support association		
Conversations with the spouse	Support from spouse	
Support in daily routines by spouse		
Fellowship with spouse		
Support in daily routines by family and friends	Support from family and friends	
Keeping in touch with family and friends		
Parents´ mental health is cared for by close family and friends		
Get crisis assistance	Mental health services available to parents	Mental wellbeing support
Availability of adult psychiatry services		
Mental health services provided outside the hospital		
Meeting with other workers at the hospital		
Support from the early interaction (Vavu) team		
Starting psychotropic medication	Ways to support parents´ mental health	
Guiding parents about the effects of trauma on the mind and body		
Processing events with healthcare professionals		
Assessment of parents' mental health by healthcare professionals		
Support from healthcare professionals for parents to take care of basic needs		

### 4.5 Ethical Considerations

The research was conducted in accordance with good scientific principles (Finnish National Board on Research Integrity, TENK 2023). Ethical approval was obtained from the regional medical ethics committee of the Wellbeing Services of the country of North Ostrobothnia (EETTMK 42/2022). The cooperation contract on participant recruitment with the National Association for Families with Premature Infants was made before the recruitment began. A participant information sheet and an EU GDPR‐compliant privacy notice were provided to all parents, allowing them time to consider their participation before providing written consent to participate. Before participating, parents had the opportunity to ask questions, and they were reminded that participation was voluntary and they could withdraw their participation at any time before the results were published without any consequences. Before the interviews, researchers spoke with parents to inform them that difficult memories may come to mind, so they could interrupt or take a break at any time. The researcher reminded parents that they can get more help with difficult feelings by contacting a healthcare provider if needed.

### 4.6 Rigour and Reflexivity

The research team consisted of six female members: two PhD students in nursing science, three professors (nursing science, neonatology and child psychiatry) and a PhD in psychology. In this multidisciplinary research team, all members provided their own scientific perspective and expertise. The interviews were collected by one of the authors (H.S.), a researcher who had experience working as a nurse with children and families and experience in collecting qualitative interview data from sensitive target groups. The researcher has not treated the families participating in the study. The other researchers possessed strong methodological and substantive expertise, and they were closely involved in planning the data collection. In the data analysis, several researchers (H.S., E.T., T.P.) participated to ensure reliability. The process of the research was described as accurately as possible.

## 5 Findings

### 5.1 Participant Characteristics

A total of 15 parents, 12 mothers (80%) and three fathers (20%), aged 22–42 years (mean 32.9 years), whose infant had been treated in the NICU in Finland, participated in the study. The study participants were from four university hospitals and two central hospitals. Of the participants, 53% of parents reported that their infant was treated in a single‐family room. Seven of the parents also had previous children, while eight parents reported that this child was their first. Two parents had an earlier experience of intensive care with their previous child. The gestational age at birth ranged from 24 to 38 weeks. Eight of the parents had a caesarean section, and seven had a vaginal delivery, one of which was a twin pregnancy. The average duration of a NICU stay was 48 days (range 3–124 days).

### 5.2 Parents' Experiences of Psychosocial Support in the NICU

According to the results, parents' experiences of psychosocial support consisted of four main categories: (1) supporting parenting; (2) strengthening the sense of security; (3) social support; and (4) mental wellbeing support (Figure 1).

**FIGURE 1:**
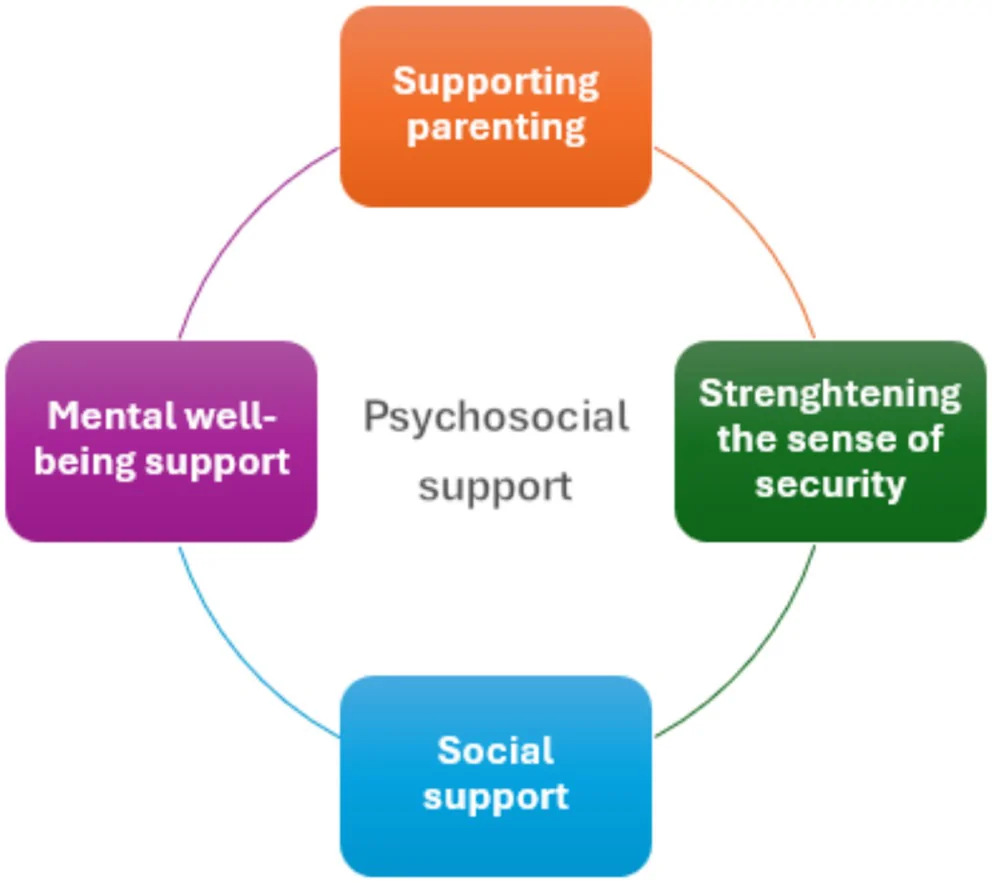
The parents' experiences of psychosocial support in the neonatal intensive care unit. [Colour figure can be viewed at wileyonlinelibrary.com]

### 5.3 Supporting Parenting

Supporting parenting consists of three categories: support from healthcare professionals at different stages of care, environmental support for parental presence and increasing parental participation in care.

#### 5.3.1 Support From Healthcare Professionals at Different Stages of Care

Parents felt that preparing them for their infant's admission to the intensive care unit in advance supported them. Parents said that a prenatal visit to the NICU made the spaces more familiar. Also, meeting with the NICU doctor and with the peer‐group facilitator in advance gave parents information about what was going to happen. Psychological support initiated before birth also supported parents

For example, the meeting with the doctor was very good, so when we went to the section, we knew what is possible going forward. (Mother 2, pair interview)

It was important for the parents to get support from healthcare professionals during the infant's care. Parents wanted to get support and information about early interaction methods they could use in the NICU. Singing, reading and skin‐to‐skin contact were ways to interact with the infant, and these activities made parents feel they were important. Also, healthcare professionals' support in strengthening early interaction was important, such as placing the infant next to the parents on the bed when it was possible. Parents described how nurses supported early interaction despite the infant being in the NICU while the mother got treatment in a different department. Nurses could show pictures of the infant or transported the mother on the bed to see the infant, when she could not go to see the infant due to her own condition.

That skin to skin, there was a lot of encouragement for it and… so there was a lot of that then. That matter was clearly brought out… and it is important, and I felt it was important to myself. (Mother 7)

The parents described how the guidance for infant care supported them. The nurses guided the parents in infant care, such as handling, diaper changing, washing and breastfeeding or bottle‐feeding. Receiving repeated concrete help with infant care was described as being important. Nurses supported parents when parents felt insecure. Nurses made sure that parents felt comfortable with the infant before they left them alone in the room.

They teach us by hand how to give milk, change diapers, wash or handle the baby every day. (Mother 10)

Discussions with healthcare professionals about the infant's health status were important to the parents and reduced their uncertainty. Parents wanted to receive information about their infant's health and to understand the goals of the care. They reported feeling relieved when healthcare professionals said that they were not concerned about their infant's condition. Mothers who were themselves being treated in the hospital highlighted opportunities to get information about their infant's health.

It helped when you knew about things and that you were on the top of things, like medication, nutrition and other things, when you were there all the time on the spot and chatted with doctors and nurses. (Mother 10)

Guiding parents to notice progress in their infant's health status supported the parents. Healthcare professionals celebrated with parents even when the infants achieved just small goals or when there was progress in the infant's health. For example, healthcare professionals could give greeting cards when an infant achieved 1 month of age. If parents visited at home during neonatal intensive care, nurses told parents they could call the NICU anytime.

In the hospital, they celebrated every kilogram and every goal there was. I learned that the little things give positive energy to me, and I can also celebrate these little things. (Mother 1)

It was important to prepare parents for infant discharge. Discussions with NICU nurses and public health nurses at the maternity and child health clinic were good ways to prepare parents for the discharge. Parents highlighted the importance of reducing treatments, like test weighing and monitoring, before the infant was discharged from the NICU. It was meaningful to increase parental participation in infant care before discharge, because it supported survival at home and reduced the parents' needs for support at home.

They encouraged us to call into the NICU and said they can give advice and guide us, but I didn't feel the need, because I had already been taking care of the baby for the last few weeks in the intensive care unit. (Mother 10)

#### 5.3.2 Environmental Support for Parental Presence

Parents described the environment that supports parental presence as meaningful. The possibility of privacy and of being in a single‐family room were important to parents. Comfortable rooms, with their own bathroom and decent beds, gave both parents the possibility to be in the NICU around the clock. Healthcare professionals supported parents' privacy by providing privacy screens or curtains and closing the doors if possible.

At least we got a lot of privacy; actually, the nurses pulled the door shut often when they, for example, noticed I started to read. (Mother 12)

Parents emphasised the importance of a calming space with coffee facilities. This calming space in the NICU gave parents the opportunity to take a breath and spend little time in silence.

As you hear, when I tell you how important a place that parents' lounge has been to us. It was a place where we could rest a while during that two and half weeks. (Father 5, pair interview)

An environment that considers the needs of parents was described as supportive. New and well‐equipped facilities and services, such as shops, cafes or restaurants, supported parents in hospital. Parents also appreciated the opportunity for visitors, like grandparents or the infant's siblings, in the NICU.

These were brand new premises, and there were restaurants and cafes and shopping facilities, so everyday life started quite well there. (Mother 12)

Healthcare professionals guided parents through the NICU environment. Nurses explained the significance of the NICU alarms to the parents, which helped they understand which alarms are critical. Several parents described how they reacted to every alarm at the beginning of treatment, but then they learned when it's necessary to react and when it's not.

Of course, when the pulse went down the first time, or things like that happened, we were scared, but it comforted us when the nurse was there the whole time and when they explained what these things mean. (Mother 1)

#### 5.3.3 Increasing Parental Participation in the Care

Parents described participatory practices and working methods as supporting them. It was important that healthcare professionals let parents take care of their infant and encouraged them to participate. Parents felt supported when they and healthcare professionals jointly cared for the infant.

They were all so lovely. We took care of the baby together, and they took us as part of the care team. Being part of the team was very important to us. (Mother 6, pair interview)

Having the opportunity to participate in the care planning and decision making was experienced as valuable by the parents. Healthcare professionals took parents into account during a doctor's visit and asked their opinions on different aspects of care.

We were really taken into account; that started during the first days when they asked our opinions about the baby's situation and what we think about the amount of milk, and we were truly involved in it. (Mother 10)

Healthcare professionals supported parents by considering their individual wishes and needs regarding care, such as wishes for the schedule, breastfeeding and holding the infant in one's arms.

She had heard me crying and struggling, so she got a pillow and asked if I will bring both of the babies to the breast at the same time, and then I wondered if I could really touch my children, and she said that yes, you can; so, then both babies breastfed for the first time, and I remember this nurse, she returned the children to us. (Mother 5, pair interview)

### 5.4 Strengthening the Sense of Security

The main category, strengthening the sense of security, consisted of two categories: emotional support and safety‐enhancing practices.

#### 5.4.1 Emotional Support

Compassionate behaviour of nurses and doctors supported parents, and human and warm encounters were important to them. It was meaningful that the healthcare professionals and the atmosphere were calm in chaotic situations.

In some way, it tells something about these people, about their humanity, that I still miss these primary nurses and doctors. (Father 6, pair interview)

There were really calm doctors, who didn't engage in useless chatter. (Mother 10)

Parents preferred the nurses' good social skills. It was significant to have lighter conversations and to talk about everyday things and family situations with healthcare professionals. Parents also pointed out how humour with nurses was supportive.

We were there a long time, and they discussed other things with us, such as our life and about nurses´ children and dogs and cats, and that was really important. That there was always someone to talk with about other things than baby. (Father 6, pair interview)

Nurses were described as understanding the parents' situation and having a good sense of the situation. The nurses' ability to read and understand the parents was supportive. Especially primary nurses understood parents' experiences, and some of the parents described how primary nurses always found the right words and how primary nurses’ support was one of the most important things.

The nurses may have a big influence; my baby had the kind of primary nurses who understood the things I went through. (Mother 1)

Primary nurses had done much more than just cared for the baby. So I think their support was one most of the important things. (Mother 2, pair interview)

Parents needed support from healthcare professionals when they experienced guilt. Healthcare professionals were able to support parents by stressing that it's not the parents' fault that the infants need neonatal intensive care.

They tried to drum it into my head that it wasn't my fault, you couldn't have done anything. (Mother 1)

Parents highlighted that the most important feeling is that you and your family are well cared for in the NICU.

Fortunately, the child was also taken care of extremely well, and so were we, the parents. (Father 6, pair interview)

#### 5.4.2 Safety‐Enhancing Practices

Parents preferred safety‐enhancing practices. Parents cared for the infant more independently when conditions allowed, but they still highlighted how important it was to feel that help is immediately available if needed. Also, the sense of security increased when parents felt that more professional conversation support was available if needed.

Even though I take care of children a lot myself, if something had happened, someone would have been there right away. (Mother 7)

A clear division of responsibilities between the healthcare professionals and the parents was important. Parents described it as relieving when healthcare professionals clearly communicated their responsibility for the infant's medical treatment and monitoring.

The nurses said that they take care of the monitors, and your mission is to take care of kangaroo care and feeding; everything related to the equipment is the nurses' responsibility. (Mother 12)

It increased parents' sense of safety when healthcare professionals had sufficient time with parents. It was important to feel that the nurses had time to come help, answer questions or explore the infant's care together. Parents wanted to feel that they were not alone, and they described nurses' presence was supportive.

I felt, all the time, that the nurses had time to respond, and they had time to help and time to look at things together with us. (Mother 12)

All the nurses who cared for the baby did so great, they were also a present for me as a parent. (Mother 3)

It was considered important that social workers had time to counsel and fill out forms together with parents and answer their questions, when they helped parents in applying for social benefits.

It was really relieving when you didn't need to find out these things for yourself; we were able to do just what they said, and she had time, and she responded to us. (Mother 12)

That the same nurses took care of the infant was described as meaningful. Parents reported that it was relieving to see familiar nurses coming on for the next shift and how they felt supported when primary nurses were on the shift.

It was really important that it would be the same people as much as possible, from the beginning until the child was discharged. (Father 6, pair interview)

Competent healthcare professionals made parents feel safe and increased their confidence. When healthcare professionals had a strong experience, they could support parents by giving examples, such as how the infants' treatment usually goes. It was important for the parents to feel that the healthcare professionals did their best to help the infant.

I had strong confidence in the healthcare professionals and felt that there was nothing to worry about. (Mother 11)

### 5.5 Social Support

Social support consists of three categories: peer support, support from spouse and support from family and friends.

#### 5.5.1 Peer Support

Peer support was one of the most important things for many parents and they shared experiences with peers because they understood exactly what they felt. Parents shared experiences with roommates or other parents whose infants were in the NICU. Sharing experiences with peers was very important to both parents. Fathers reported how peer support encouraged them, and it was a relief to hear that other fathers had the same kind of thoughts. Some of the parents reported how peers became close friends and how they kept in touch face‐to‐face.

It was a fathers' own moment, and we could share fathers’ experiences then, how everything has gone and what everybody has experienced. There, you could ask questions from a paternal perspective. (Father 2, pair interview)

It was also important to obtain contact information for a peer‐support association, as this enabled parents to access peer support. Parents described how they took part in different peer‐support groups, such as hospital and associations' peer‐support groups.

Then, we got a peer‐support association's contact information. (Mother 2, pair interview)

Finally, after coronavirus, there were peer‐support groups organised that I visited a few times. There were other parents who were in the NICU, so I visited there a few times, I think that was good. (Mother 1)

The use of peer‐support on internet was also described. Some parents read empowering peer stories on the internet, because it was interesting for them to read about how things had gone for other families. Some of the parents described how it was a relief to read about families who have had infants in the NICU, but everything is okay now.

On Instagram, I started to follow many accounts who had premature babies. I have watched and listened to how things are going for them. (Mother 9)

Participation in peer‐support groups on internet was also described and some of the parents reported how peers on internet became close friends and they keep in touch on social media and with phone calls and messages.

I have that well‐functioning WhatsApp group. There, I get peer support, and it helps to stay in everyday life. (Mother 4)

#### 5.5.2 Support From Spouse

Support from a spouse was described as important. Parents felt that conversations with their spouse were supportive. They had face‐to‐face conversations, but also phone calls if both parents could not be in the NICU. Especially mothers described the importance of processing events with their spouse.

There has been lot of talking about it at home, and we have vented our emotions. (Mother 1)

Spouses' support for each other in daily routines was also meaningful. Parents did some light activities with their spouse, such as going to the hospital's cafeteria or outdoors. Some parents mentioned that their spouse supported their wellbeing by taking care of their basic needs, such as meals.

Those have been really important to me, and to both of us, that we took some pastries in the hospital's cafeteria with coffee, or that we went outside, it helped us endure. (Mother 2, pair interview)

Physical and mental presence of the spouse was meaningful. Especially mothers described how important it was that the father could be out of work and be in the NICU with the mother and infant. Spouses calmed and grounded each other. It was important to get security from one's own spouse.

That you (spouse) were off work then, you carried me through; he cooked for us, heated the food and made sure I ate. (Mother 6, pair interview)

My spouse is quite good at grounding me. I am the kind of person where thoughts go down the wrong paths, so he knows how to bring me back. (Mother 1)

Parents described fellowship with their spouse as supportive. It was important to feel that spouses were a team and in the same situation. Parents also encouraged each other. They might have said to each other, ‘*We'll get through this*’.

One big thing to us was that we reminded each other that we are in this situation together, and we have each other, and we could talk things over with each other. (Mother 2, pair interview)

#### 5.5.3 Support From Family and Friends

Parents got support in daily routines from family and friends. Family members and friends supported parents by bringing food, meal tickets or doing the shopping for parents. They also reminded parents to go out. Grandparents helped by taking care of the other children and pets, that supported parents, because that way they could spend more time in the NICU.

I had someone who said that now you should go for a short walk. (Mother 7)

My parents took good care of our older children, so of course it helped us. (Mother 11)

Keeping in touch with family and friends was also an important way to deal with the matter. Especially mothers highlighted the importance of conversations with close friends and family, such as their own mother, siblings and friends.

I received so much help from my mum, so I feel that she helped me carry on. (Mother 7)

Family and friends take care of parents’ mental well‐being. Some parents reported that friends and family noticed that they needed more psychological support, which helped them to have stronger support quickly.

We have an extended circle of family and friends, and everybody was interested, so I was able to go through the event again and again. I always explained it again, and I felt it helped. (Mother 12)

### 5.6 Mental Wellbeing Support

The parents' experiences of mental wellbeing support consisted of two categories: mental health services available to parents and ways to support parents' mental health.

#### 5.6.1 Mental Health Services Available to Parents

Parents reserved different mental health services during the neonatal intensive care. Some parents reported getting crisis assistance, meeting with a crisis worker once or on several occasions. They highlighted the value of meeting a crisis worker early, particularly during the first week in the NICU. These meetings were described as helpful in processing what had happened. Some parents attended the sessions individually, while others participated together with their spouse.

Our first meeting with the crisis worker was a kind of debriefing; it was quite good, it helped to organise our thoughts. (Mother 2, pair interview)

Availability of adult psychiatry services was important to the parents. They took part in adult psychiatry services, where they had conversations with different healthcare professionals, such as psychiatrists, psychologists and nurses.

I was completely overwhelmed, or rather, I started thinking about it really intensely, so I was referred to adult psychiatry and got to talk to someone. (Mother 8)

The use of mental health services provided outside the hospital during the infant's intensive care stay was described. Few parents had psychotherapy in progress, and they felt it was supportive to talk with their own therapist. Fathers also reported receiving support through occupational health services.

I went to talk to my own therapist immediately. (Mother 1)

Meetings with other hospital staff were also experienced as supportive. Concrete advice from social workers on how to take care of various matters was perceived as relieving, and parents appreciated not having to find things out on their own in already challenging situations. It was also considered important that social workers provided information to both parents

There was a social worker who came to introduce these basic benefits and gave us a contact form. (Mother 10)

…and she checked that we knew how to apply for everything… She had the possibility to help with these. (Mother 2, pair interview)

Few parents reported how they felt supported after meeting a music therapist, a hospital chaplain or a third‐sector worker. However, participants reported receiving less support of this kind.

I met the music therapist. It's been researched that it is useful to the baby, so that was quite interesting. (Mother 8)

Support from the early interaction team (Vavu) was important. It was helpful to meet the early interaction support team immediately during the first week in the NICU and to meet with these workers weekly. Parents felt it was good that the support continued for a long time, even after the infant's discharge from the NICU. These meetings with the early interaction (Vavu) team increased positive early interaction between parents and the infant.

Meetings with the early interaction support team, and support like that, continued over a long period, so it helped for a long time. (Mother 11)

#### 5.6.2 Ways to Support Parents` Mental Health

Parents described different ways to support their mental health during infant intensive care. Some parents reported that they started psychotropic medication during their infant's neonatal intensive care. The medication supported the parents' mental wellbeing and adequate sleep.

Sleeping problems started immediately, so I got medication for that. (Father 6, pair interview)

Guiding the effects of trauma on the mind and body was important. When healthcare professionals gave information about what happened when people face something traumatic, it helped parents to understand their thoughts and emotions and helped parents to understand that their reactions were normal with traumatic experiences.

It helped when we didn't just talk about the crisis, but we also talked about the trauma. I learned to associate my symptoms with the trauma. (Mother 6, pair interview)

Parents felt it was relieving to process events together with different healthcare professionals. Some of the parents said it was also good to talk with healthcare professionals without their own spouse and described processing emotions with healthcare professionals. Healthcare professionals could normalise parents' emotions, which helped them understand that these emotions are normal in a challenging situation.

I received good psychological support. I don't know how I would have survived if I hadn't had someone to talk to about things. (Mother 7)

Because I had the care contact, I was able to speak, so I didn’t feel shame. (Mother 7)

It was important that the assessment of parents' mental health was conducted by healthcare professionals. Parents highlighted the importance of NICU professionals' knowledge of their mental health conditions. Conversations with familiar nurses about their wellbeing were seen as a good way to gain information about their mental health. Stronger support for parents was needed when they required it. Parents highlighted the importance of timely psychological support and the availability of information about various psychological support services. Providing support to all parents, including those who did not request it, was considered important.

We were like a family. They knew more about our life than my own family or friends; I couldn't keep in touch with them, but doctors and nurses knew about our condition. (Mother 6, pair interview)

I was actually able to go to the psychiatric services pretty quickly; I talked with a nurse and that was really important to me. (Mother 11)

Support from healthcare professionals for parents to take care of basic needs was meaningful. For example, the nurses reminded parents to eat and to do outdoor activities.

It was one thing that I always forgot: I forgot to order a meal, and finally the nurses suggested that it would be okay if we put the meal on a standing order, so I wouldn't have to worry about it. (Mother 9)

## 6 Discussion

This study provides new insights on the parents' experiences of psychosocial support in the NICU. Many previous studies focused only on preterm and low birthweight infants and their families (Franck and O'Brien 2019) or just mothers (Kestler‐Peleg et al. 2022; Segre et al. 2014). By including full‐time infants and perspectives from both maternal and paternal caregivers, this study provides a more comprehensive understanding of psychosocial support in Finnish NICUs. Despite the distress of NICU parents being recognised and some evidence‐based practices to reduce this distress having been developed, there are still significant gaps in parent support services (Ginsberg et al. 2023). Based on this study, all parents reported having individual experiences of psychosocial support, and various types of psychosocial support were identified. In this study, parents described the supportiveness they experienced when they received supporting parenting, strengthening the sense of security, social support and mental wellbeing support. The results show that there are many ways to offer psychosocial support for NICU parents. Although the study included parents whose infants were in very different situations in terms of disease severity, all parents described a need for psychosocial support.

Based on this study, supporting parenting at different stages of neonatal intensive care is vital to the parents, and parents want to participate in the care of the infant. They prefer when healthcare professionals involve them, and they take care of the infant together. The parents' roles and the need to improve parents' partnership in the care of infants is recognised, but it's still limited in many NICUs (Franck and O'Brien 2019), which was also reflected in this study. Parents highlighted the importance of supporting parents in taking care of their infants. Many parents described how they could participate as much as they wanted in their infant's care, but there were still parents who felt that participation was limited. Parents felt useful when they had early interaction with the infant, for example, when they kept the infant in kangaroo care. Early interaction, such as kangaroo care, between parents and the infant supports the infant's health and development (Boundy et al. 2016); but in this study, it was also meaningful to the parents. Parents also highlighted the possibility of taking part in decision making. This finding has been reported in previous studies, particularly in Family‐Centred Care (FCC) studies (Gooding et al. 2011). In this study, parents described how parental support encompasses both concrete help and advice, as well as participatory practices and working methods. In previous studies, it has been recognised that also unit facilities, such as supportive visitation policy, privacy and the possibility of getting a family billet near the hospital, can support parents, but parents rated these infrastructural needs as low (Adama et al. 2022), which is the opposite result to the one in this study. In this study, parents emphasised the importance of a functioning environment. Most parents wanted to be in the NICU around the clock, so they preferred single‐family rooms and good services, such as the opportunity to buy food.

The study identified various ways to support parents' sense of security. This study confirms the results of previous studies that when an infant is in the NICU, parents need empathetic encounters and emotional support (Bry and Wigert 2019; Loewenstein et al. 2019). Parents described how healthcare professionals' good interaction skills, ability to read parents and friendly and human encounter supported them. Also, Adama et al. (2022) have reported in a previous study that healthcare professionals' emotional intelligence can support parents' wellbeing in the NICU. This study adds to the knowledge that fathers and parents of full‐term infants in the NICU also need empathic encounters and support. The fathers emphasised the need to be heard and recognised as parents. Some described the value of having opportunities to talk even without their spouse present. Addressing these paternal needs in the NICU is essential. Healthcare professionals can also increase a parent's sense of security by setting a clear division of responsibilities and by building trust between parents and healthcare professionals.

Social support was important to the parents. The empowering effects of peer support on the parents have been recognised (Pascual et al. 2024). The results of this study showed that parents use various forms of peer support, including digital options, and find these empowering. In addition, spousal support, particularly the presence of a partner, was described as especially important by mothers. The importance of spousal support for mothers has also been recognised (Kestler‐Peleg et al. 2022; Yang et al. 2017). This study provides new insight into fathers' experiences of their spouses' support. Fathers described how they shared things with their spouses, and how that increased fellowship. This has been identified in fathers whose child has been seriously ill (Koivula et al. 2019). Based on this study, parents, including fathers, valued spousal support most and also relied on family and friends. The inability of family and friends to visit the NICU, due to pandemic‐related or hospital restrictions, was experienced as stressful. In previous studies, it has been reported that an unlimited visit policy for family and friends in the NICU increased family‐centred care and may support parents (Axelin et al. 2022). This study provided information on the importance of support in daily routines and how it enabled parents to stay in the NICU.

Parents brought up the meaning of mental wellbeing support. They appreciated the quickly available mental health services. Parents can benefit from mental health services, such as conversational help (Bry and Wigert 2019). In this study, parents highlighted both the diversity of available mental health services and their fragmented nature. Parents described using mental health services provided by the hospital as well as services outside the hospital. In the future, examining parents' access to mental health services is warranted, as some parents described long waiting times, complicated care pathways, and the psychological burden of repeatedly explaining their situation to multiple professionals. Parents also described diverse ways of supporting their mental well‐being during their infant's NICU treatment. They highlighted the importance of the NICU professionals' assessment of parents' mental health. In this study, parents also wanted to get information about trauma symptoms, which is a new aspect. They described how it increased understanding and helped to process events. Trauma‐informed care might increase parents' sense of security, but further study is needed (Sanders and Hall 2018). Previously, the benefits of trauma‐informed care for parents have been identified and it has been recommended that family‐centred care be updated to include trauma‐informed care.

### 6.1 Strengths and Limitations of the Work

This study strengthens the previous knowledge base on the topic, but it also provides new knowledge from the perspective of those who have been excluded in several previous studies, such as fathers and parents of infants born full‐term. The reliability of the study was assessed by examining credibility, transferability, dependability and confirmability (Lincoln and Guba 1985). The systematic and transparently described analysis process enhances the dependability of the findings. The use of verbatim citations strengthens the credibility and supports the link between the data and the findings. Data saturation, defined as the point at which no new information would be generated (Kyngäs et al. 2019), was being reached. Although complete saturation is rarely achievable in qualitative research, key themes were consistently present in the data, suggesting that additional interviews would likely have yielded only marginal new information. The research data was deep and rich, but we recognise there is a small sample size of the parents collected from one country. However, this number of interviewees has been shown to be sufficient for saturation when the studied group is homogeneous (Hennink and Kaiser 2022). Although both fathers and mothers participated in the study, and their infants' conditions and lengths of NICU stay varied, a common factor for all families was the infant's need for intensive care. It is possible that more severe infant illness increases parents' need for psychosocial support. However, in this study, all parents described multiple forms of psychosocial support that they found helpful, regardless of the severity of the infant's condition or the duration of treatment.

In this study, parents were recruited through the peer support association's website. The researchers do not know how many parents who met the inclusion and exclusion criteria were reached by the research call, but it is possible that parents who were more interested in the topic participated in the study, whereas those who still felt mentally unwell may not have participated. Parents who had lost an infant were excluded from the study, which may have led to the results lacking suitable psychosocial support methods for these situations.

The data still includes respondents from several central and university hospitals. Most of the participants were mothers, so their views might be more strongly represented than those of the fathers. Although the data were collected from NICUs, the results can be transferred, to some extent, to other parents with seriously ill children (Lechner et al. 2024).

## 7 Conclusion

Based on this study, parents have individual psychosocial support needs when their infant is in the NICU. Parents receive psychosocial support from various providers, but the level and quality of that support vary. The NICUs have different practices on what kind of support is offered to parents and how or how quickly support can be obtained. In some NICUs, parents received crisis support without being asked, while in others, parents had to wait a long time for psychological support, even though they felt they needed it. The NICU also employed a variety of staff, including crisis workers, but the services offered to parents varied.

It's important to recognise these different ways to support parents and provide current psychosocial support. Psychosocial support should include parenting support, strengthen parents' sense of security, facilitate social support, such as peer support, and provide parents with timely and adequate mental health support in NICUs. Many parents emphasised the importance of peer support, yet such support was available in only a few hospitals. Expanding access to peer support should therefore be considered in the future.

Healthcare professionals recognising and understanding parental psychosocial support can increase parental satisfaction and improve the wellbeing of the families both in the NICU and after discharge. The parents hoped for an encounter that would strengthen safety, so it would be important to investigate the applicability and effectiveness of trauma‐informed care at the NICU in the future. Also, the experiences of the psychosocial support of fathers and siblings, who were excluded from previous studies, should be further investigated to obtain a complete picture. In addition, new types of psychosocial interventions should be developed and tested to evaluate their effectiveness on families' psychological burden and wellbeing.

## Author Contributions

Conceptualization: Hanna Seppänen, Ulla Sankilampi, Krista Koivula, Ilona Luoma and Tarja Pölkki. Formal analysis: Hanna Seppänen, Eeva Talus and Tarja Pölkki. Funding acquisition: Hanna Seppänen and Tarja Pölkki. Investigation: Hanna Seppänen. Methodology: Hanna Seppänen, Ulla Sankilampi, Krista Koivula, Ilona Luoma and Tarja Pölkki. Supervision: Tarja Pölkki and Ulla Sankilampi. Validation: Hanna Seppänen, Eeva Talus and Tarja Pölkki. Writing the manuscript: Hanna Seppänen, Tarja Pölkki, Ulla Sankilampi, Krista Koivula, Ilona Luoma and Eeva Talus.

## Funding

This work was supported by The Finnish Cultural Foundation, the Päivikki and Sakari Sohlberg Foundation and The Foundation for Paediatric Research.

## Conflicts of Interest

The authors declare no conflicts of interest.

## Supporting information


**Data S1:** Supporting Information.

## Data Availability

The data that support the findings of this study are available on request from the corresponding author. The data are not publicly available due to privacy or ethical restrictions.
